# Asymptomatic unruptured intracranial aneurysms in the older people

**DOI:** 10.1007/s41999-018-0122-7

**Published:** 2018-10-29

**Authors:** Sang Woo Ha, Pahn Kyu Choi, Ji Eun Oh, Jung Soo Park, Hyun Goo Kang

**Affiliations:** 1 Department of Neurosurgery, Chosun University School of Medicine and Hospital, Gwang-ju, 61453 South Korea; 2 Department of Neurology, Chosun University School of Medicine and Hospital, Gwang-ju, 61453 South Korea; 3 Department of Nursing Service, Chosun University School of Medicine and Hospital, Gwang-ju, 61453 South Korea; 4 Department of Neurosurgery, Chonbuk National University Hospital, Jeonju, 54907 South Korea; 5 Department of Neurology, Research Institute of Clinical Medicine Chonbuk National University, Biomedical Research Institute of Chonbuk National University Hospital, 20 Geonji-ro, Deokjin-gu, Jeonju-Si, Jeonbuk-do 54907 South Korea

**Keywords:** ASPECT ratio, Asymptomatic, Coronary artery disease, Older people, Unruptured aneurysm

## Abstract

**Purpose:**

Unruptured intracranial aneurysm commonly occurs in the older people. Because the rupture risk increases with age, the factors associated with aneurysms might be different according to age. We aimed to evaluate unruptured intracranial aneurysm characteristics in healthy, symptom-free older patients.

**Methods:**

Patients who visited the health examination center of two regional university hospitals and underwent computed tomography angiography between March 2001 and March 2017 were included. The putative aneurysm risk factors were identified; the aneurysm size and shape were determined by CT angiography, and measuring the ASPECT and dome/neck ratios. All images were interpreted independently by a neurosurgeon and a neurologist for improving size measurement accuracy.

**Results:**

The unruptured intracranial aneurysm prevalence was 2.23% and 2.75% in the patients aged ≤ 60 and > 60 years, respectively. Among the younger group, female sex [odds ratio (OR), 1.85; *P *= 0.002], age (OR, 1.05; *P *< 0.001), hypertension (OR, 1.88; *P *< 0.001), coronary artery disease (OR, 0.26; *P *< 0.001), smoking (OR, 2.04; *P *< 0.001), and stroke family history (OR, 1.36; *P *= 0.047) were independently associated with aneurysm; anterior communicating artery aneurysms were the largest. Among the older group, female sex (OR, 1.76; *P *= 0.005), hypertension (OR, 2.54; *P *< 0.001), coronary artery disease (OR, 0.27; *P *< 0.001), and stroke family history (OR, 1.94; *P *= 0.003) were independently associated with aneurysm; internal carotid artery aneurysms were the largest.

**Conclusions:**

The factors related to unruptured intracranial aneurysm formation varied by age, and coronary artery disease protected against aneurysm formation regardless of age. The factors affecting unruptured intracranial aneurysm formation are different according to age and aneurysm location.

## Introduction

Recently, older people ratio changes particularly increased the risk of cerebrovascular disease, which is common in the older people. Older patients often have a variety of comorbid diseases, and therefore require different treatments than young patients. Consequently, it is critical to identify age-specific risk factors and disease characteristics in older patients clearly.

Unruptured intracranial aneurysm (UIA) is caused by secondary vessel wall bulging due to various factors, including high blood flow pressure on the blood vessel, blood vessel shape, and genetic factors [1, 2]. UIA is a cerebrovascular disease that commonly occurs in the older people and has a prevalence of 0.8–2.8% [3–6]. Fortunately, the recent advancement of non-invasive neuroimaging technology, such as computed tomography angiography (CTA) and 3-Tesla magnetic resonance angiography (MRA), has increased the detection rate and accuracy [7]. Intracranial aneurysm ruptures cause sudden subarachnoid hemorrhages, which lead to high morbidity and fatality [6]. Particularly, the rupture risk increases with age. Therefore, UIA in the older people needs appropriate treatment. However, the characteristics, general consensus, and optimal treatment of UIA in the older people remain unknown. Also, in case of health check-ups conducted, people who are completely healthy, not those who have concomitant diseases, voluntarily visit clinics to enforce it. Concomitant disease is more common over 60 years old, and they are often diagnosed aneurysms for disease-related symptoms rather than aneurysms found by health check-ups.

This study aimed to identify the UIA characteristics in healthy, symptom-free older patients and examine if there are differences in the risk factors or shape and location of aneurysms.

## Methods

### Patient population

**Fig. 1:**
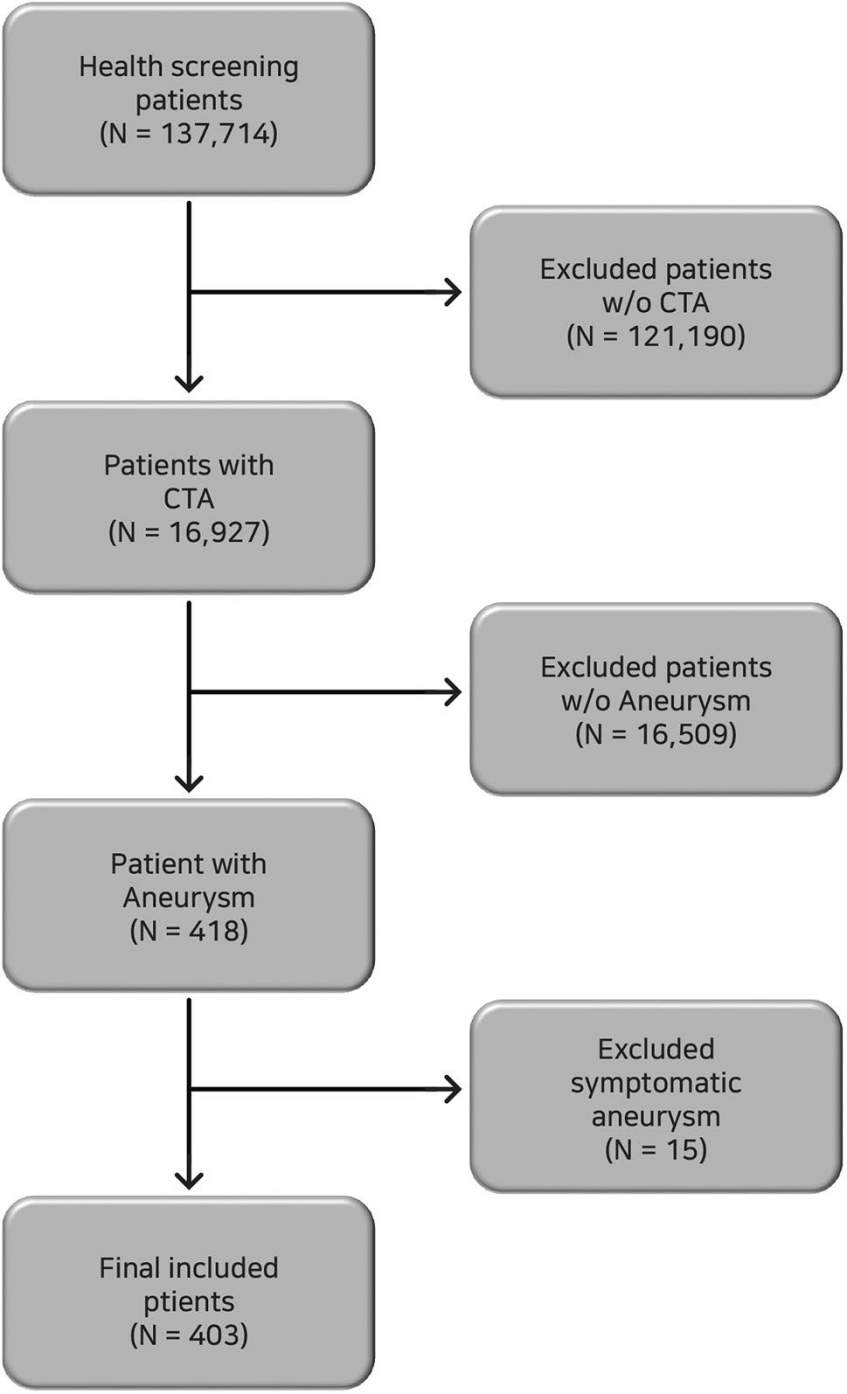
Illustration of the selection of the study participants with unruptured intracranial aneurysms. CTA, computed tomography angiography

Patients who visited the health examination centers of the university hospitals in two regions from March 2001 to March 2017 were included. All participants were healthy at the time of examinations and did not receive any public advertisement; they independently selected and visited the hospitals. This study consecutively enrolled patients who underwent brain CTA and were diagnosed with UIA. Patients who had fusiform, mycotic, or traumatic aneurysms or already-treated (surgically or endovascularly) aneurysms before CTA were excluded. Moreover, patients who had frequent headaches identified from the medical interview were also excluded, because they had a possibility of developing symptomatic aneurysms (Fig. 1). The local institutional review board approved this study. The requirement to obtain informed consent was waived because of the retrospective nature.

### Data collection and definition

The participants’ demographic information and clinical data were obtained by retrospectively reviewing items in the checklists, which were filled up by the participants in advance. The results of laboratory tests conducted during physical examination were also reviewed. These items included hypertension, diabetes mellitus (DM), coronary artery disease (CAD), current smoking, and alcohol consumption, which are the putative risk factors of aneurysms. Hypertension was defined as at least one blood pressure measurement of ≥ 140/90 mmHg at rest or hypertension medication use. DM was defined as either a blood glucose level of > 200 mg/dL for at least 2 h after an oral glucose challenge or a fasting blood glucose level of > 126 mg/dL or DM medication use [8]. Current smoking and alcohol consumption were defined as the presence of current smoking and alcohol consumption habits. CAD was defined as the presence of a CAD diagnosis by a cardiologist and CAD medication use or a history of percutaneous coronary intervention or bypass surgery.

### Imaging analysis

CTA was conducted using Toshiba Aquilion ONE, multi-slice CT machine (Toshiba, Japan). CTA images were obtained with 64-slice CT scanners using a helical acquisition of 0.625-mm section thickness from the aortic arch through the circle of Willis after injection of 80–110 mL of a contrast medium. First, contrast-free images and contrasted CTA images were obtained 60 s after non-ionic contrast medium injection (80 mL; ≥ 300 mg/mL) using an automatic injector at a rate of 4 mL/s and an 18-G needle into the antecubital vein. The slice thickness was 0.625 mm across all series, and the machine settings were 100 kV, 200 mAs, FOV 220 mm, and 512 × 512 matrix.

The aneurysm size was measured using three-dimensional reconstruction images. The neck width was defined as the virtual line of aneurysms separated from the parental artery. The maximum height was defined as the distance from the aneurysm neck to the dome tip. The maximum width was defined as the maximum aneurysm width, perpendicular to the height. The aneurysm size was then determined as the largest value among these measurements. When there were multiple aneurysms, it was defined as the largest value of an aneurysm. The ASPECT ratio was obtained by dividing the previously measured maximum height measured by the neck width, and the ratio indicates the aneurysm slenderness [9]. The dome/neck ratio was estimated by dividing the maximum aneurysm width by the neck width, and the value presents the degree of aneurysm swelling [2].

The aneurysm location was classified into the distal internal carotid artery (ICA), middle cerebral artery trunk (MCA), MCA bifurcation, anterior cerebral artery, anterior communicating artery (Acom), posterior communicating artery (Pcom), and posterior circulating artery (including vertebral, basilar, posterior cerebral, and anterior/posterior inferior cerebellar arteries) [5]. Furthermore, aneurysms at the end or branch of the blood vessel that experiences hemodynamic stress were defined and classified as end-wall artery aneurysms. All CTA images were interpreted independently by a neurosurgeon (H.S.K.) and a neurologist (H.W.R.). If there was a discrepancy between the original report and reader’s interpretation or if the presence of an aneurysm was equivocal, another neurologist (H.G.K.) reviewed the records to reach a consensus.

### Statistical analysis

The prevalence of UIA among the patients who underwent CTA for 17 years was calculated. The participants were first divided into two groups by age: older (> 60 years) and younger (≤ 60 years) groups. Afterward, each group was divided into sub-groups of participants with and without aneurysms. Univariate and multivariate analyses were conducted to evaluate the factors associated with aneurysm formation by age. To avoid variable selection caused by spurious correlations, only variables showing a potential association (*P *< 0.1) in the univariate analysis were included in the multivariate logistic regression model.

Moreover, the demographics, risk factors, and aneurysm characteristics of the participants with aneurysms were recorded by age. We also examined if the aneurysm characteristics were different by aneurysm location and age. For comparisons, we performed Pearson *χ*^2^ test and Student’s *t* test for categorical and continuous variables, respectively. A two-sided *P *< 0.05 was considered statistically significant. All statistical analyses were performed using SPSS 21 (IBM Corp., Armonk, NY).

## Results

During the study period, 137,714 patients underwent routine health examinations, and 16,927 (12.3%) of them underwent CTA. Among those who underwent CTA, 403 (2.38%) had asymptomatic unruptured aneurysms, and 193 (47.9%) of them were women. The mean age of aneurysm patient was 57.22 ± 8.56 years.

### Factors related to aneurysms in the younger group

Out of the 16,927 patients, 12,214 (72.1%) were aged ≤ 60 years, and 273 of them (2.23%) had aneurysms. Those aged ≤ 60 years with aneurysms comprised more women (43.2% versus 36.6%; *P *= 0.026), and their mean age was higher than that of the participants without aneurysms (52.64 ± 5.76 versus 51.45 ± 6.02; *P *= 0.001) and with lower CAD rates (7.3% versus 18.6%; *P *< 0.001). Moreover, many of them were smokers (28.9% versus 21.4%; *P *= 0.003) and had a greater stroke family history (33.7% versus 24.4%; *P *< 0.001). However, the body mass index (BMI) and ESR of the participants with aneurysms were somewhat lower than those of the participants without (Table 1). The multivariate logistic regression analysis showed that female sex [OR, 1.85 (1.20–2.73); *P *= 0.002], age [OR, 1.05 (1.02–1.08); *P *< 0.001], hypertension [OR, 1.88 (1.37–2.58); *P *< 0.001], CAD [OR, 0.26 (0.15–0.43); *P *< 0.001], smoking [OR, 2.04 (1.45–2.87); *P *< 0.001], family history of stroke [OR, 1.36 (1.01–1.85); *P *= 0.047], and ESR [OR, 0.97 (0.96–0.99); *P *= 0.005] were independently associated with aneurysms (Table 2).

**Table 1:** Baseline characteristics of the study participants with and without aneurysms who were aged ≤ 60 and > 60 years

	≤60 Years			>60 Years		
	NR (*n *= 11,941)	AN (*n *= 273)	*P* value	NR (*n *= 4583)	AN (*n *= 130)	*P* value
Female sex	4376 (36.6)	118 (43.2)	0.026	2005 (43.7)	75 (57.7)	0.002
Age	51.4 ± 6.1	52.6 ± 5.7	0.001	66.9 ± 5.0	66.8 ± 4.6	0.712
Hypertension	3189 (26.7)	86 (31.5)	0.078	2124 (46.3)	76 (58.5)	0.007
CAD	2226 (18.6)	20 (7.3)	< 0.001	1422 (31.0)	24 (18.5)	0.003
Diabetes mellitus	1208 (10.1)	25 (9.2)	0.063	949 (20.7)	28 (21.5)	0.818
Smoking	2559 (21.4)	79 (28.9)	0.003	421 (9.2)	9 (6.9)	0.377
Alcohol drinking	6911 (57.9)	155 (56.8)	0.716	1672 (36.5)	47 (36.2)	0.939
F/Hx of hypertension	5227 (43.8)	129 (47.3)	0.252	1571 (34.3)	51 (39.1)	0.241
F/Hx of CAD	1726 (14.5)	44 (16.1)	0.44	446 (9.7)	11 (8.5)	0.629
F/Hx of stroke	2911 (24.4)	92 (33.7)	< 0.001	909 (19.8)	40 (30.8)	0.002
BMI	24.3 ± 2.9	23.9 ± 3.1	0.027	24.4 ± 2.8	24.1 ± 2.8	0.229
Hb	14.5 ± 1.5	14.3 ± 1.3	0.048	14.1 ± 1.4	13.8 ± 1.5	0.012
Hct	43.2 ± 3.9	42.6 ± 3.6	0.006	42.1 ± 3.8	41.2 ± 4.0	0.005
ESR	13.1 ± 10.6	11.6 ± 9.2	0.044	17.7 ± 14.2	17.4 ± 12.7	0.781
hS-CRP	0.1 ± 0.2	0.1 ± 0.2	0.894	0.2 ± 0.3	0.1 ± 0.1	< 0.001
PT INR	1.0 ± 0.6	1.0 ± 0.6	0.5	1.0 ± 0.1	1.0 ± 0.1	0.864
aPTT	28.2 ± 2.1	27.9 ± 2.0	0.08	27.9 ± 2.3	27.6 ± 1.8	0.069
Albumin	4.2 ± 0.3	4.2 ± 0.3	0.038	4.1 ± 0.3	4.1 ± 0.3	0.784
Na	142.0 ± 2.1	142.2 ± 2.2	0.055	142.4 ± 2.4	142.7 ± 2.1	0.228
K	4.1 ± 0.3	4.1 ± 0.3	0.289	4.1 ± 0.4	4.1 ± 0.4	0.242
BUN	13.1 ± 3.5	13.0 ± 3.4	0.723	14.2 ± 4.4	14.5 ± 4.5	0.417
Cr	0.9 ± 0.2	0.8 ± 0.2	< 0.001	0.9 ± 0.3	0.9 ± 0.4	0.344
HbA1c	5.7 ± 0.8	5.6 ± 0.6	0.58	6.0 ± 0.9	5.9 ± 0.9	0.615
TC	194.9 ± 35.5	190.3 ± 34.3	0.032	187.5 ± 36.5	185.6 ± 36.8	0.567
TG	129.0 ± 82.3	123.5 ± 73.5	0.275	122.7 ± 73.9	112.9 ± 53.5	0.134
HDL	54.3 ± 13.9	54.6 ± 15.9	0.703	53.2 ± 13.5	54.3 ± 13.9	0.377
LDL	122.3 ± 31.1	119.4 ± 30.3	0.121	117.0 ± 32.0	116.7 ± 32.5	0.925
TSH	2.9 ± 3.6	3.1 ± 2.8	0.34	3.0 ± 4.0	2.8 ± 1.9	0.58
fT4	1.3 ± 0.2	1.3 ± 0.2	0.73	1.3 ± 0.2	1.3 ± 0.2	0.901
Uric acid	5.5 ± 1.4	5.2 ± 1.4	0.002	5.3 ± 1.4	5.2 ± 1.4	0.787

Values are presented as the number of patients (%) or means (SDs). *P* values were calculated using Pearson *χ*^2^ test or Student’s *t*-test, as appropriate

*AN* patients with aneurysm, *BMI* body mass index, *CAD* coronary artery disease, *CRP* C-reactive protein, *ESR* erythrocyte sedimentation rate, *F/Hx* family history, *Hb* hemoglobin, *Hct* hematocrit, *HDL* high-density lipoprotein, *LDL* low-density lipoprotein, *NR* normal patients, *TC* total cholesterol, *TG* triglyceride, *TSH* thyroid-stimulating hormone

*P* value of < 0.05 was considered statistically significant

**Table 2:** Factors associated with the occurrence of aneurysms in the patients aged ≤ 60 and ≥  61 years

	Crude OR (95% CI)	*P* value	Adjusted OR (95% CI)	*P* value
≤60 years				
Female sex	1.32 (1.03–1.68)	0.026	1.85 (1.20–2.73)	0.002
Age	1.04 (1.01–1.06)	0.001	1.05 (1.02–1.08)	< 0.001
Hypertension	1.26 (0.97–1.64)	0.078	1.88 (1.37–2.58)	< 0.001
CAD	0.34 (0.22–0.54)	< 0.001	0.26 (0.15–0.43)	< 0.001
Smoking	1.49 (1.14–1.95)	0.003	2.04 (1.45–2.87)	< 0.001
F/Hx of stroke	1.58 (1.22–2.03)	< 0.001	1.36 (1.01–1.85)	0.042
BMI	0.95 (0.92–0.99)	0.027	0.97 (0.92–1.02)	0.234
ESR	0.98 (0.97–1.00)	0.044	0.97 (0.96–0.99)	0.005
Albumin	0.62 (0.39–0.97)	0.038	0.76 (0.73–1.33)	0.341
TC	0.99 (0.99–1.00)	0.032	0.99 (0.99–1.00)	0.092
Uric acid	0.87 (0.79–0.95)	0.002	0.99 (0.87–1.12)	0.948
> 60 years				
Female sex	1.75 (1.23–2.49)	0.002	1.76 (1.18–2.63)	0.005
Hypertension	1.63 (1.14–2.32)	0.007	2.54 (1.67–3.86)	< 0.001
CAD	0.50 (0.32–0.78)	0.003	0.27 (0.16–0.46)	< 0.001
F/Hx of stroke	1.79 (1.23–2.63)	0.002	1.94 (1.26–2.97)	0.003
hs–CRP	0.26 (0.07–0.99)	< 0.001	0.21 (0.05–0.89)	0.034

*P* values present the results of the multivariable logistic regression

Variables with *P *< 0.1 in the univariate analysis were entered into the multivariate analysis model

*BMI* body mass index, *CAD* coronary artery disease, *CI* confidence interval, *ESR* erythrocyte sedimentation rate, *F/Hx* family history, *TC* total cholesterol, *CRP* C-reactive protein, *F/Hx* family history, *OR* odds ratio

*P* value of < 0.05 was considered statistically significant

### Factors related to aneurysms in the older group

There were 4713 (27.8%) patients who were aged > 60 years, and 130 (2.75%) of them had aneurysms. Those with aneurysms also comprised more women (57.7% versus 43.7%; *P *= 0.026); however, the mean age was not different between the patients with and without aneurysms (66.82 ± 4.59 versus 66.99 ± 5.04; *P *= 0.712). Many of them had hypertension (58.5% versus 46.3%; *P *= 0.007) and stroke family history (30.8% versus 19.8%; *P *= 0.002); however, the number of the patients with CAD was relatively small (18.5% versus 31.0%; *P *=0.003). The hs-CRP level was somewhat lower in those with aneurysms (0.11 ± 0.13 versus 0.16 ± 0.25; *P *< 0.001) (Table 1). The multivariate logistic regression analysis showed that female sex [OR, 1.76 (1.18–2.63); *P *= 0.005], hypertension [OR, 2.54 (1.67–3.86); *P *< 0.001], CAD [OR, 0.27 (0.16–0.46); *P *< 0.001], stroke family history [OR, 1.94 (1.26–2.97); *P *= 0.003], and hs-CRP level [OR, 0.21 (0.05–0.89); *P *= 0.034] were independently related to aneurysms (Table 2).

### Comparisons of the patients with aneurysms by age

The younger group had higher rates of posterior circulation aneurysms and smoking and alcohol consumption, while the older group had a higher proportion of female participants and rate of multiple aneurysms; they also had more underlying diseases, such as hypertension, CAD, and DM (Table 3). The aneurysms were larger in the older group than in the young group (4.49 ± 2.23 versus 3.88 ± 1.35; *P *= 0.001). However, there was no difference in the ASPECT (1.43 ± 0.67 versus 1.49 ± 0.66; *P *= 0.393) and dome/neck ratios (1.50 ± 0.68 versus 1.56 ± 0.62; *P *= 0.423) between them. The aneurysm locations were not generally different; however, Pcom and posterior circulation aneurysms were more common in the younger group (Table 3).

**Table 3:** Comparison of the characteristics of the patients with aneurysms who were aged ≤ 60 and > 60 years

	≤60 years (*n * = 273)	>60 years (*n * = 130)	*P* value
Age (year)	52.6 ± 5.8	66.8 ± 4.6	
Female sex	118 (43.2)	75 (57.7)	0.007
Multiple aneurysms	16 (5.9)	16 (12.3)	0.025
Posterior circulation	17 (6.2)	2 (1.5)	0.014
Hypertension	86 (31.5)	76 (58.5)	< 0.001
CAD	20 (7.3)	24 (18.5)	0.001
Diabetes mellitus	25 (9.2)	28 (21.5)	0.001
Smoking	79 (28.9)	9 (6.9)	< 0.001
Alcohol drinking	155 (56.8)	47 (36.2)	< 0.001
F/Hx of hypertension	129 (47.3)	51 (39.2)	0.130
F/Hx of CAD	44 (16.1)	11 (8.5)	0.036
F/Hx of stroke	92 (33.7)	40 (30.8)	0.558
BMI	24.0 ± 3.1	24.1 ± 2.8	0.718
Hb	14.4 ± 1.4	13.8 ± 1.5	< 0.001
Hct	42.6 ± 3.6	41.2 ± 4.0	< 0.001
ESR	11.6 ± 9.2	17.4 ± 12.7	< 0.001
hS-CRP	0.1 ± 0.2	0.1 ± 0.1	0.259
PT INR	1.0 ± 0.1	1.0 ± 0.1	0.044
aPTT	28.0 ± 2.0	27.6 ± 1.8	0.065
Albumin	4.2 ± 0.3	4.1 ± 0.3	0.039
Na	142.2 ± 2.2	142.7 ± 2.1	0.072
K	4.1 ± 0.3	4.1 ± 0.4	0.606
BUN	13.0 ± 3.4	14.5 ± 4.5	< 0.001
Cr	0.8 ± 0.2	0.9 ± 0.4	0.276
HbA1c	5.6 ± 0.6	5.9 ± 0.9	0.002
TC	190.3 ± 34.3	185.6 ± 36.8	0.213
TG	123.5 ± 73.5	112.9 ± 53.5	0.100
HDL	54.6 ± 15.9	54.3 ± 13.9	0.823
LDL	119.4 ± 30.3	116.7 ± 32.5	0.416
TSH	3.1 ± 2.8	2.8 ± 1.9	0.385
fT4	1.3 ± 0.2	1.3 ± 0.2	0.021
Uric acid	5.2 ± 1.4	5.2 ± 1.4	0.866
Aneurysm characteristics			
Size of aneurysm	3.9 ± 1.4	4.5 ± 2.2	0.001
ASPECT ratio	1.5 ± 0.7	1.4 ± 0.7	0.393
Dome/neck ratio	1.6 ± 0.6	1.5 ± 0.6	0.423
Location of aneurysm			
ICA (*n *= 178)	119 (43.6)	59 (45.4)	0.439
MCA (M1 and M2) (*n *= 27)	17 (6.2)	10 (7.7)	
MCA bifurcation (*n *= 54)	34 (12.5)	20 (15.4)	
ACA (*n *= 28)	19 (7.0)	9 (6.9)	
Acom (*n* = 55)	36 (13.2)	19 (14.6)	
Pcom (*n *= 42)	31 (11.4)	11 (8.5)	
Posterior circulation (*n *= 19)	17 (6.2)	2 (1.5)	

Values are presented as the number of patients (%) or means (SDs). *P* values were calculated using Pearson χ^2^ test or Student’s *t*-test, as appropriate

*ACA* anterior cerebral artery, *Acom* anterior communicating artery, *BMI* body mass index, *CAD* coronary artery disease, *CRP* C-reactive protein, *ESR* erythrocyte sedimentation rate, *F/Hx* family history, *Hb* hemoglobin, *Hct* hematocrit, *HDL* high-density lipoprotein, *ICA* internal carotid artery, *LDL* low-density lipoprotein, *MCA* middle cerebral artery, *Pcom* posterior communicating artery, *TC* total cholesterol, *TG* triglyceride, *TSH* thyroid-stimulating hormone

*P* value of < 0.05 was considered statistically significant

### Difference according to aneurysm location

The aneurysm location was different between the two age groups (Table 4). In the younger group with aneurysms, the Acom aneurysm size was relatively larger than the mean aneurysm size. The ASPECT ratios showed significant differences by location; the ICA aneurysms showed the lowest ratio, and the Acom and MCA bifurcation aneurysms showed higher ratios. The dome/neck ratio also revealed a significant difference by location; the ICA aneurysms showed the lowest ratio, and the Acom and MCA bifurcation aneurysms showed higher ratios. The BMI also varied by location; those with posterior circulation or Acom aneurysms had high BMIs. All MCA bifurcation aneurysms and approximately half of the Acom aneurysms were located in the end-wall artery.

**Table 4:** Characteristics of the patients with aneurysms who were aged ≤ 60 and > 60 years according to artery location

	≤60 years							
	ICA (*n *= 119)	MCA (*n* = 17)	MCA bifurcation (*n *= 34)	ACA (*n *= 19)	Acom (*n *= 36)	Pcom (*n *= 31)	Posterior circulation (*n *= 17)	*P* value
Size	3.8 ± 1.0	4.2 ± 1.6	3.7 ± 1.3	3.5 ± 1.0	4.4 ± 2.2	4.2 ± 1.7	4.2 ± 1.7	0.173
ASPECT ratio	1.3 ± 0.5	1.6 ± 0.8	1.7 ± 0.6	1.6 ± 0.8	2.0 ± 0.8	1.3 ± 0.5	1.5 ± 0.8	< 0.001
Dome/neck ratio	1.4 ± 0.4	1.8 ± 0.9	1.7 ± 0.6	1.8 ± 0.7	1.9 ± 1.0	1.5 ± 0.5	1.6 ± 0.5	< 0.001
BMI	23.2 ± 2.7	24.7 ± 3.6	24.9 ± 3.6	23.6 ± 3.0	25.2 ± 2.9	23.7 ± 3.3	25.2 ± 2.6	0.002
End-wall artery	0 (0)	2 (11.8)	34 (100)	1 (5.3)	19 (52.8)	4 (12.9)	4 (23.5)	< 0.001

	> 60 years							
	ICA (*n *= 59)	MCA (*n *= 10)	MCA bifurcation (*n *= 20)	ACA (*n *= 9)	Acom (*n *= 19)	Pcom (*n *= 11)	Posterior circulation (*n *= 2)	*P* value
Size	4.8 ± 2.7	4.4 ± 2.5	4.1 ± 1.3	3.4 ± 0.8	4.0 ± 1.8	4.8 ± 1.6	5.1 ± 2.9	0.593
ASPECT ratio	1.2 ± 0.6	1.2 ± 0.6	2.0 ± 0.7	1.6 ± 0.7	1.5 ± 0.7	1.3 ± 0.5	1.6 ± 0.4	0.001
Dome/neck ratio	1.4 ± 0.4	1.3 ± 0.4	1.7 ± 0.7	1.6 ± 0.7	1.7 ± 0.9	1.5 ± 0.3	2.0 ± 1.3	0.048
BMI	23.8 ± 2.8	24.3 ± 3.2	24.3 ± 2.9	23.3 ± 3.2	25.1 ± 2.6	24.1 ± 2.8	22.9 ± 4.0	0.672
End-wall artery	0 (0)	3 (30)	20 (100)	1 (11.1)	10 (52.6)	1 (9.1)	2 (100)	< 0.001

Values are presented as the number of patients (%) or means (SDs). *P* values were calculated using Pearson χ2 test or Student’s *t*-test, as appropriate

*ACA* anterior cerebral artery, *Acom* anterior communicating artery, *BMI* body mass index, *ICA* internal carotid artery, *MCA* middle cerebral artery, *Pcom* posterior communicating artery

*P* value of < 0.05 was considered statistically significant

In the older group with aneurysms, the ICA aneurysms were the largest. The ASPECT ratio was significantly different between locations, and the MCA bifurcation aneurysms showed the highest ratio. The dome/neck ratio was also significantly different between locations, and the Acom and MCA bifurcation aneurysms showed the highest ratios. Those with Acom aneurysms had the highest BMI. The end-wall artery aneurysm ratio was similar between the two age groups.

## Discussion

The overall asymptomatic UIA prevalence was 2.38%. The UIA prevalence was 2.23% and 2.75% in the younger and older groups, respectively. The results are consistent with those of a previous study showing that the UIA prevalence was slightly higher in older patients [10]. The prevalence in this study is similar to those of previous studies [4, 7, 11]. However, the results of this study are meaningful, because this study evaluated the difference in the aneurysm occurrence by age by examining healthy asymptomatic participants, including those with small aneurysms, using CTA.

There was reportedly no significant difference in the aneurysm size measurements using CTA and MRA [12]. CTA has a disadvantage in which high-density structures, such as bony structures, can interfere with blood vessel reconstruction in image reconstruction. MRA also has several constraints; measuring the aneurysm size if it is too small or is located in the proximal part of the vessel is difficult. Therefore, high-resolution images, such as 3-TeslaMRA images, should be used [5, 12]. Moreover, it is challenging for hospitals to conduct MRA using high-resolution images owing to its high cost. Therefore, it is difficult to analyze small aneurysms. However, CTA can complement these limitations, because it allows analyses by comparing reconstruction and source images.

In the younger group, UIA was common in the women, as also reported in previous studies, and was highly associated with hypertension and active smoking [10]. Women have more inflammatory reactions due to estrogen deprivation after menopause [13]. Moreover, hypertension can lead to hemodynamic stress, which can cause UIA [5] Additionally, smoking causes inflammatory reactions that weaken the extracellular matrix of the blood vessels, resulting in aneurysms [14]. UIA was more common in the older group than in the younger group, and the ratio of multiple UIAs was also higher. It was more common in the women and was related to hypertension. However, smoking was not related to UIA occurrence in the older group. These results may have been observed, because the inflammatory reactions induced by smoking were small in the older group or the extracellular matrix of the blood vessels was already weakened too much. The aneurysm was larger with an older age, and the result supports the hypothesis that the extracellular matrix is weakened prominently in the old age. However, the aneurysm shape as assessed using the ASPECT and dome/neck ratios did not show great differences by age. Thus, blood vessel changes due to aging may not affect the shape formation of aneurysms. CAD protected against aneurysm formation regardless of age [5]. Vascular degeneration progression may then induce aneurysm formation and cause atherosclerosis in some blood vessels. These hypotheses need to be proven by analyzing aneurysm prevalence, risk factors associated with aneurysm, and atherosclerosis in many patients with CAD in the future.

UIA commonly occurred in the distal ICA regardless of age, followed by the Acom and MCA bifurcation. However, posterior circulation UIA was more commonly observed in the younger group than in the older group. Although it is difficult to explain the exact reason for such results, it is though that the generation of UIA in the posterior circulation is associated with an increased blood pressure [5] and younger patients have a higher chance of abrupt blood pressure increase. And also, the Acom UIA was the largest, and the ASPECT and dome/neck ratios were the highest among all locations in the younger group. The occurrence of aneurysms in the Acom in the younger group may pose a high rupture risk, because they are large and of the saccular type. Moreover, the patients with posterior circulation and Acom UIAs showed high BMIs, and hemodynamic factors could be important in these locations, because they are related to obesity and hypertension.

In the older group, ICA UIA was the largest; however, those with Acom UIA showed the highest BMI. Acom or MCA bifurcation UIA often occurs in the end-wall artery rather than in the side-wall artery. The end-wall artery receives much hemodynamic stress. MCA bifurcation UIA occurs more commonly in the end-wall artery than Acom UIA. However, the Acom UIA was larger. It could be because it received more hemodynamic stress, since the Acom was closer to the aorta than the MCA bifurcation, and there were fewer originating branches. Considering that the BMI was the highest in those with Acom UIA, hypertension was an important factor in its formation. Consequently, the hemodynamic factor may be very important in Acom UIA formation. Additional large-scale prospective studies on Acom UIA are needed to prove the bases of these results.

The findings of this study are meaningful, because it analyzed the factors associated with asymptomatic UIA formation in a large number of patients by age. Moreover, this study utilized CTA to identify even small aneurysms accurately, and one neurosurgeon and one neurologist checked the images for improving UIA size measurement accuracy. However, this study has several limitations. First, the possibility of a selection bias should be clarified, because the data collection and analyses were conducted retrospectively. Generally, individuals who undergo medical check-ups are healthy and can afford to do so. Therefore, this study might have excluded patients with asymptomatic UIA, which generally occurs in patients with poor health. Consequently, the study could have underestimated the prevalence. Second, this study was a cross-sectional study without long-term monitoring. However, it was difficult to monitor patients for a long duration and analyze monitoring data, because the participants irregularly visited the hospitals, which is common among patients visiting for medical check-ups, and often do not undergo examinations at the same hospital.

Asymptomatic UIA formation is associated with various factors, such as hemodynamic and degenerative factors. However, the factors related to UIA formation varied by age, and CAD protected against aneurysm formation regardless of age. Moreover, Acom UIA was highly affected by hemodynamic stress; it could pose a high rupture risk, because Acom UIA was of the saccular type and larger than those in the other locations. The factors affecting UIA formation were different by age and UIA location. It would be critical to determine the UIA treatment direction considering these factors.
